# Chemotherapy increases the prevalence of radiotherapy-related trismus in head and neck cancer patients: A systematic review and meta-analysis

**DOI:** 10.4317/jced.61385

**Published:** 2024-04-01

**Authors:** Marcela-Maria-Fontes Borges, Cássia-Emanuella-Nóbrega Malta, Reverton-Soares Ribeiro, Edson-Luiz Cetira-Filho, José-Fernando-Bastos de Moura, Lievin-Matos Rebouças, Fábio-Wildson-Gurgel Costa, Paulo-Goberlânio-de Barros Silva, Mario-Rogerio-Lima Mota

**Affiliations:** 1 Department of Dental Clinic, Division of Oral Pathology, Faculty of Pharmacy, Dentistry and Nursing, Federal University of Ceará, Fortaleza, Ceará, Brazil; 2 Department of Dentistry, Unichristus, Fortaleza, Ceará, Brazil; 3 Hospital Haroldo Juaçaba, Ceará Cancer Institute, Fortaleza, Ceará, Brazil

## Abstract

**Background:**

To evaluate the influence of chemotherapy on the prevalence of trismus in irradiated head and neck cancer patients.

**Material and Methods:**

This systematic review guided by PRISMA-2020 and registered in PROSPERO (CRD42021255377) screened 963 articles in 7 scientific-databases (PubMed, Lilacs, Livivo, Scopus, Embase, Web of Science, EBSCO) and 3 grey-literature databases (Open Grey, Google Scholar, ProQuest) and eight articles were included for qualitative synthesis, meta-analysis (combined odds ratio, inverse variance method plus random effects), heterogeneity analysis (I² and Tau²), one-of-out evaluation and publication bias analysis (Eggs’ and Begg’s tests) (RevMan®, *p*<0.05). The Newcastle-Ottawa Quality Assessment Scale Cohort Studies was used to assess the risk of bias (RoB). The classification assessment, development, and recommendations (GRADE) approach was used to assess the certainty of evidence.

**Results:**

The eight articles evaluated 1474 patients treated with chemoradiotherapy and 858 patients treated with radiotherapy. Five articles had low RoB, and three had high RoB. Chemoradiotherapy significantly (*p*=0.0003) increased the prevalence of trismus (OR=2.55, 95% CI = 1.53-4.23) compared to radiotherapy, with significant (*p*=0.010) but low heterogeneity (I²=59%;Tau²=0.29). There was no significant risk of publication bias, one-out analysis showed no significant difference between studies, and GRADE showed a moderate level of evidence. Trismus was directly associated to worse quality of life.

**Conclusions:**

The incidence of trismus increases when chemotherapy is combined with radiotherapy for head and neck cancer, which negatively impacts the quality of life.

** Key words:**Radiotherapy, Chemoradiotherapy, Head and Neck Neoplasms, Trismus, Quality of Life.

## Introduction

Currently, RCT is the first line of adjuvant or palliative treatment for head and neck tumors. The combination of RT (3D or IMRT) with chemotherapy has significantly increased the life expectancy of these patients Guan’ *et al*. (1). However, numerous adverse effects are also increased in RCT compared to RT (2-4).

The effects of treatment with RT, QT, or with chemoradiotherapy (QRT) have a high potential to generate direct damage to the tissues of the oral cavity, thus entailing a significant negative impact on the quality of life of patients, and can lead to xerostomia, radiation caries, oral mucositis, osteoradionecrosis, oral infection, trismus, stomatitis, loss of taste, periodontal and nutritional disease (5,6).

RT uses electromagnetic ionizing energy, which interacts directly and indirectly with target tissues causing cellular damage and inflammation (7). Patients who have RT alone or in conjunction with QT for treatment of SCC have as standard the daily regimen or the total number of fractions of radiation treatment used a dose of 1 treatment of 2 Gy per day (8,9).

Besides adverse effects such as neutropenia, thrombopenia, anemia, infections, nausea, vomiting, mucositis, dermatitis, neurotoxicity, xerostomia, chemoradiotherapy is also associated with muscle fibrosis, speech difficulties, dysphagia, and trismus are the most common. These effects can predispose patients to problems such as inadequate nutrition and may even lead to the interruption of cancer treatment (6,10,11).

The maximum interincisal opening (MIO) in the healthy population ranges from 36-55 mm, measurements less than 35 mm are considered trismus (12), and it has been described that the association of concomitant radiotherapy with chemotherapy significantly increases this incidence (13). Trismus, characterized as a limitation of mouth opening, which can often interfere with the patient’s daily life, prevents basic activities of daily living such as eating, drinking, laughing, and talking (14-16) is strongly associated with muscle fibrosis post-radiotherapy (17). Radiation on masticatory muscles leads to decreased mandibular movements and can also lead to temporomandibular dysfunction (12,18).

Recently, a systematic review described the time course of RT-induced trismus. The incidence of trismus is considerably high in the first six months after initiation of radiotherapy (44.1%) and reduces very slightly even after 3 to 10 years of treatment has been completed (32.6%). Being one of the most severe late sequelae of radiotherapy treatment risk factors such as previous surgery, previous mouth opening limitation, radiation dose, and probably associated chemotherapy (19), RT-related trismus directly impacts food intake and quality of life in patients with head and neck tumors (20-22).

Since the nature of radiotherapy-related trismus is inflammatory in origin (fibrosis of the masticatory muscles) and most chemotherapies systemically increase systemic inflammatory status (23), this study aims to conduct a systematic review with meta-analysis with the main question: Patients with head and neck tumors treated with chemoradiotherapy vs. radiotherapy alone have a high incidence of trismus or masticatory difficulty?

## Material and Methods

This study was registered with a registration number CRD42021255377from the International Prospective Register of Systematic Reviews (PROSPERO) and adhered to the Preferred Reporting Items for Systematic Reviews and Meta-Analyses (PRISMA-2020) checklist (Supplement 1) (http://www.medicinaoral.com/medoralfree01/aop/jced_61385_s01.pdf), (24).

-Search strategy

A systematic review was conducted to answer the following question: “In Patients with head and neck tumors the treatment with chemoradiotherapy compared to radiotherapy increase the incidence of trismus or masticatory difficulty?” elaborated using the PECOS strategy:

Population (P): Patients with head and neck tumors. Exposition (E): Patients treated with chemoradiotherapy. Comparison (C): Patients treated with radiotherapy without chemotherapy. Outcome (O): Incidence of trismus or masticatory difficulty. Study design (S): Cross-sectional, case controls, and cohorts.

A specific search strategy was developed for each database using the descriptors “Trismus,” “Mastication,” “Chemoradiotherapy” and “Chemoradiotherapy, Adjuvant”. Appropriate truncations and word combinations were selected and adapted for each database search. Additional information on the search strategies is provided in (Supplement 2) (http://www.medicinaoral.com/medoralfree01/aop/jced_61385_s02.pdf), (24).

-Inclusion criteria

Cross-sectional studies and cohort studies (prospective and/or retrospective) evaluating the prevalence of trismus or masticatory difficulty during chemoradiotherapy; studies carried out in humans without the restriction of age, sex, ethnicity, publication time, or language, and studies with interventional and control groups (radiotherapy).

-Exclusion criteria

Case report studies, systematic reviews, studies that did not have groups to compare results (radiotherapy), duplicate and/or studies that did not report results after the end of the research; articles whose description of the research follow-up data were incomplete or had inadequately described outcomes.

-Information sources

The research was conducted at Medline via PubMed (1992 to 2020), Lilacs (1985 to 2021), Livivo (1981 to 2020), Scopus (1997 to 2021), Embase (1997 to 2020), Web of Science (1997 to 2021), EBSCO (2006 to 2020). Grey literature was investigated, and Open Grey (no data screened), Google Scholar (first 300 records: 1995 to 2021), and ProQuest (2004 to 2019) were included. A manual search was also carried out in the references of the selected articles. The search included all articles published on or before June 4, 2023, with no time restrictions.

-Selection of studies

The selection was completed in two phases. In phase 1, two reviewers (MMB and RSR) independently reviewed the titles and abstracts of all electronic database citations. Phase 1 was performed using a web application for systematic reviews (Rayyan®, Qatar Computing Research Institute, Doha, Qatar) (25). Articles that did not meet the inclusion criteria were excluded. In phase 2, the same reviewers independently applied the inclusion criteria to the articles’ full texts. One examiner (CENM) critically assessed the reference list of the selected studies. Any disagreement was resolved when the two authors reached an agreement. When they did not reach a consensus, the third and fourth authors (LECF and PGBS) participated in the final decision. PGBS performed the statistical analysis.

-Data collection process

One author (PGBS) extracted data from the selected studies, and a second author (LECJ) cross-checked all the obtained information. Any discordance between the two authors was debated until when a consensus was reached. A third author (FWGC) made the final decision when the two authors failed to reach an agreement.

-Variables

The study variables were the following surgical complications: 1) trismus incidence, 2) quality of life scores. Only the first outcome was appropriated to perform a meta-analysis, and the second outcome was qualitatively evaluated.

-Bias risk assessment and study quality

The Newcastle Ottawa Scale (NOS) assessment items were used to determine the risk of bias (RoB) in the included studies. This tool attributes scores (currently represented by a star) based on a point system among the three main domains: selection (maximum of one point per item), comparability (maximum of two points considering both items), and exposure (maximum of one point per item) It evaluates three specific domains for each study: selection (up to 4 points), comparison (up to 2 points), and exposure (up to 3 points). NOS gives a score ranging from 0–9 points, with three different RoBs interpretations: very high (0–3 points), high (4–6 points), and low risk (7–9 points). NOS has been used to assess the RoB, specifically in observational studies that can be prospective or retrospective, with evaluation criteria that involve the selected studies’ selection, comparison, and exposure process.

-Meta-analysis

The extracted data were exported to the RevMan software for meta-analysis of dichotomous data, adopting a 95% confidence level. The combined odds ratio of all studies and by the subgroup of analysis were calculated using the inverse variance method for random effects. The I² coefficient and Tau² coefficient were used to analyze heterogeneity, and Egger’s test and Begg’s test were used to analyze the risk of bias publication. The one-of-out analysis assessed each work’s influence on the overall outcome and by the subgroup of the meta-analysis.

-Quality of scientific evidence

The quality of evidence was assessed using the Grading of Recommendations, Assessment, Development, and Evaluation (GRADE) approach, reflecting the reliability in estimating the effect of the evaluated item. The GRADE profile obtained evidence certainty using the free online software GRADE pro-GDT, available at http://gdt. guidelinedevelopment.org, which was downgraded or upgraded according to the importance of some aspects (e.g., study design, bias, consistency, directness, heterogeneity, precision, publication bias, and others identified in the included studies) (26).

## Results

-Methodological characterization and study populations

**Figure 1:**
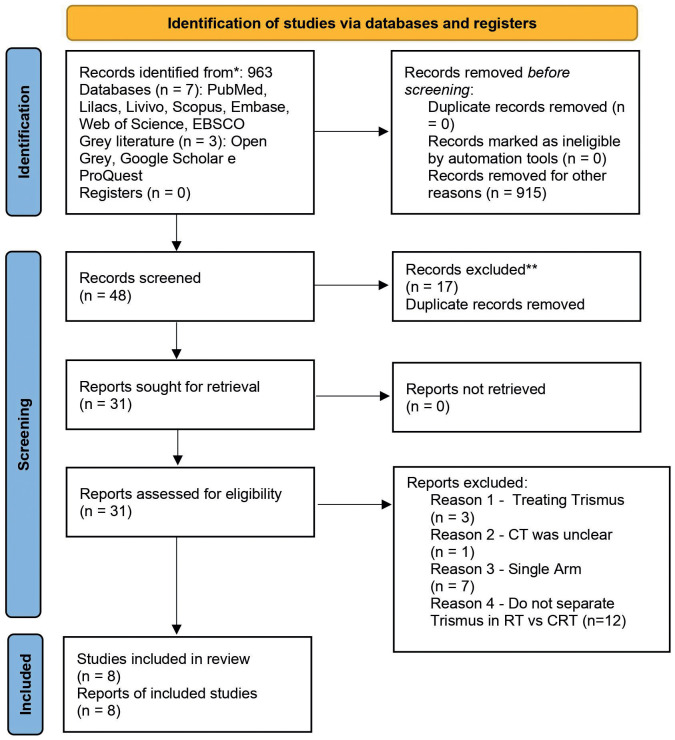
PRISMA 2020 flow diagram for new systematic reviews wich included searches of databases and registers only.

Among 963 studies evaluated in the seven scientific literature databases and the three gray literature databases, eight studies were included in this systematic review and meta-analysis, addressing a total of 2332 patients (Fig. 1). Of the eight included studies, four were cross-sectional type (16,27-29), two studies were prospective cohorts (30,31) and one was a retrospective cohort (32). Two studies were Chinese (29,31), one was North American (27), one was New Zealand (33), and the rest were European (16,28,30,32) (Table 1).

**Table 1:**
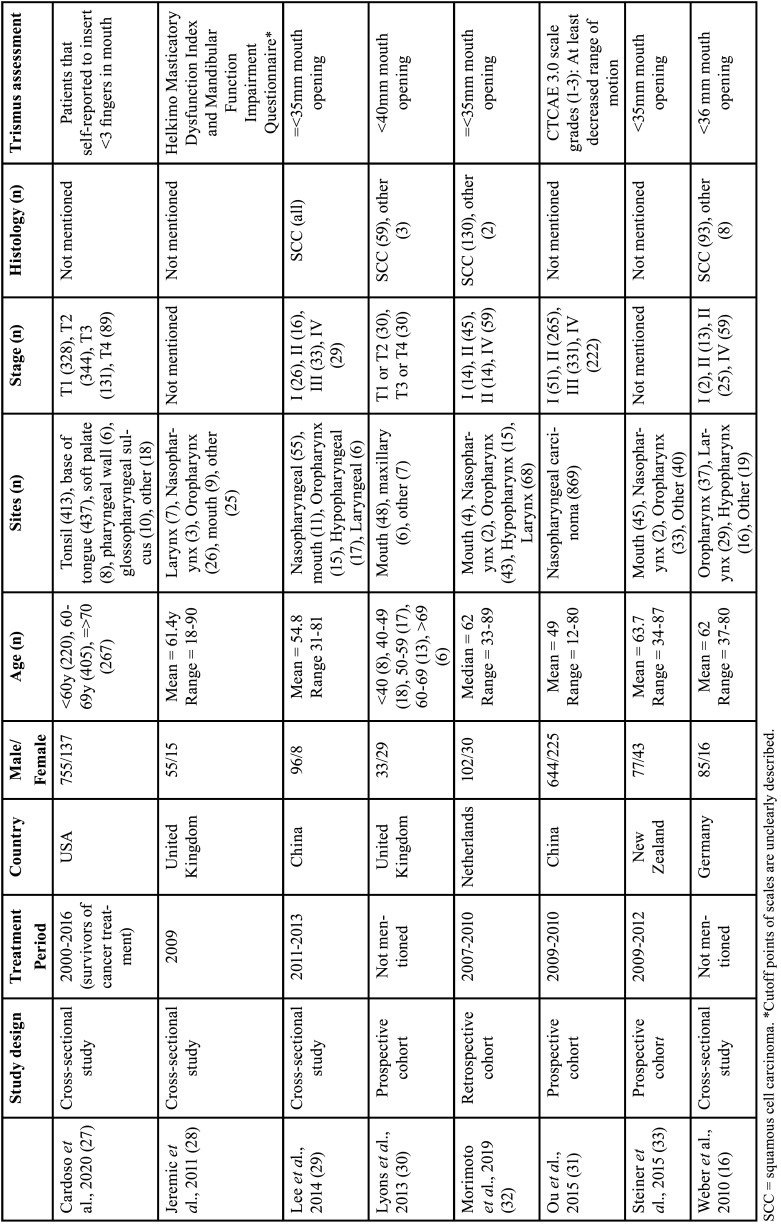
Population and methodologic assessment of trismus of studies included in systematic review.

Only (30) and (16) did not mention the study period. The oldest study with included patients was Cardoso (27) that included cancer survivors from 2000 to 2016; the others actively included patients from 2007 to 2013. The number of included patients ranged from 62 (30) to 892 (27), with a mean of 293 and a median of 112 patients per study. In all papers, the proportion of males was higher than females, and the age ranged from 12-90 years, with most patients being between 40-70 years of age (Table 1).

The most common irradiated tumor sites were the oropharynx (16,27,28,32,33), followed by mouth (30) and nasopharynx (29,31) only included patients irradiated to the nasopharynx (Table 1).

Two studies (28) did not mention tumor staging, one study (27) included most patients with T1 and T2 tumors and four studies (16,29,31,32) had patients with T3/T4 or stage III/IV tumors and one study (30) paired 30 T1/T2 tumors and 30 T3/T4 tumors (Table 1).

Four studies (27,28,31,33), did not mention the histology of the irradiated tumors, one study (29) evaluated only patients with SCC and three (16,30,32) evaluated predominantly patients with SCC (Table 1).

The most common methodology for assessing trismus was using metric scales (16,29,30,32,33) with cut-off points described in Table 1. (27) used a scale in which trismus was considered patients who could introduce less than three fingers into the mouth, (31) used the CTCAE adverse effects scale, including patients with trismus those who had at least self-reported decreased mandibular movement (scale scores 1 to 3) and Jeremic (28) used the Helkimo Masticatory Dysfunction Index scale to assess trismus and the Mandibular Function Impairment Questionnaire scale to assess masticatory efficiency. The cut-off points for diagnosing the two conditions were not clearly described in the article (28) (Table 1).

-Characterization of the therapeutic protocols of the study populations

From the eight studies included in the systematic review, four (29-32) excluded patients undergoing surgery to remove the primary tumor, but (31) also included patients undergoing nodal excision. Four studies (16,27,28,33) included patients who underwent surgery, totaling 192 patients with the removal of the primary tumor, and (27) also included 224 patients who underwent neck dissection (Table 2).

**Table 2:**
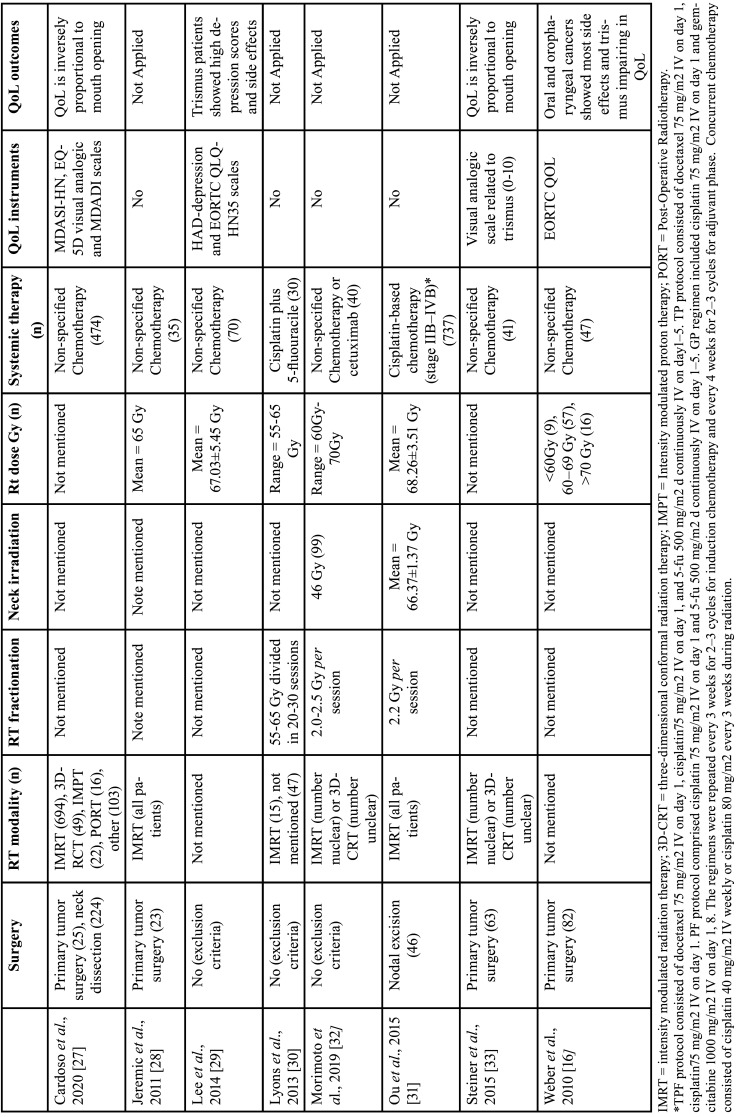
Characteristics of treatment protocol of studies included in systematic review.

Two studies (16,29) did not mention the type of radiotherapy used in the study, two (28,31) included only patients undergoing IMRT and three (27,32,33) included patients undergoing IMRT or 3D-RCT; (32) and (33) but do not make clear how many patients underwent each protocol and Lyons (30) included 15 patients undergoing IMRT and 47 undergoing other undescribed radiotherapy protocols (Table 2).

Only three studies (30-32) mentioned the fractionation of radiotherapy sessions described in Table 2. Only two studies (31,32) mentioned the radiation doses in the neck, two (27,33) did not describe the total radiation dose used, but the other studies used total doses between 60 and 70 Gy of radiation (Table 2).

Of the systemic treatments, only two (30,31) mentioned chemotherapy protocols, platinum-based, and one (32) describe the use of chemotherapies and cetuximab but does not describe chemotherapy protocols. The remaining studies did not mention the chemotherapy protocols used (Table 2).

Four studies (16,27,29,33) assessed quality of life but did not assess RT or CRT treatment outcomes. The instruments for assessing the quality of life are arranged in Table 2.

-Risk of study bias

Among the eight studies evaluated, five studies showed a low risk of bias (27-29,31,32), two showed a high risk of bias (16,30) and one showed a very high risk of bias (33) (Table 3).

**Table 3:**
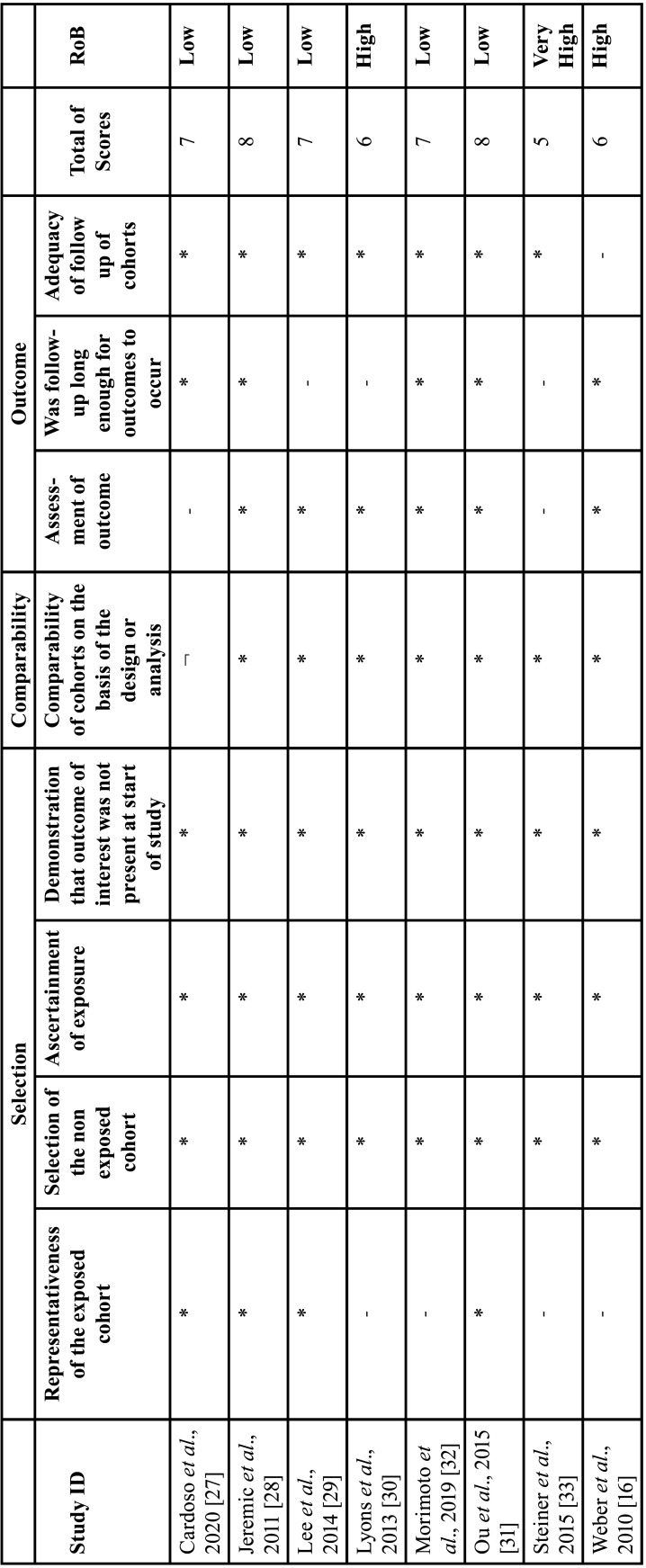
Newcasrle-Otawa scale for Risk of Bias of observational studies evaluating incidence of trismus in head and neck cancer patients receiving RCT vs. RT.

Regarding the representativeness of the exposed cohort, only four studies (27-29,31) presented this parameter. All studies selected the non-exposed cohort, ascertainment of exposure, a demonstration that the outcome of interest was not present at the start of the study, and comparability of cohorts based on design or analysis (Table 3).

One study (27) did not present an Assessment of outcome; Three (29,30,33) not present follow-up long enough for outcomes to occur and one (16) did not present adequacy of follow-up cohorts (Table 3).

-Meta-analysis and subgroup analysis

Out of the eight articles included in the systematic review, all were included in the meta-analysis. Steiner (Steiner *et al*., 2015) was the only one that evaluated the outcome of trismus comparing RT and CRT and clearly categorized patients into surgically treated and non-surgically treated (non-surgery).

Three studies (27-29,32) individually demonstrated in their studies increased prevalence of trismus in patients treated with CRT and four studies (including surgical and non-surgically treated head and neck tumors described by Steiner *et al*., 2015) (16,30,31,33) demonstrated no significant difference between the two groups studied. No study showed an increase in the prevalence of trismus in the group treated with RT.

The total patients included were 1474 patients treated with CRT and 858 patients treated with RT, and a 2.55 (CI95% = 1.53-4.23) increase in trismus was observed in the CRT patients (*p*=0.0003). There was significant heterogeneity ( I² = 59%, *p*=0.010), but this heterogeneity was low (Tau²=0.29).

Two subgroups could be assembled, a subgroup that assessed trismus using ordinal scales (27,28,31) and a subgroup with studies that assessed trismus using millimeter scales (16,29,30,32,33). When subjective methods and scales performed the analysis of trismus, a prevalence of 18.1% (331/1831) of trismus was described, and when metric methods performed the analysis of trismus, a prevalence of 22.7% (530/2332) of trismus was described. In both subgroups the prevalence of trismus was significantly higher in RCT treated patients (OR=1.93, (CI95% 1.04-3.56) and OR=2.86 (CI95% = 1.43-5.74), respectively) and there was no significant heterogeneity in the two subgroups (I² = 37%, *p*=0.210, I² = 54%, *p*=0.050, respectively) and no significant difference between the two subgroups (*p*=0.400, I² = 0%). Despite this, the prevalence of trismus by subjective methods was 0.75 (CI95% = 0.64-0.87) times lower than by the metric method (*p*<0.001).

The one-of-out analysis showed that one-to-one removal of the studies did not change the increase in the prevalence of trismus in CRT vs. RT (*p*<0.05). The funnel graph showed good distribution of the results of the articles on both sides, and only the study by (29) referring to a study taken from the database of primary platforms was out of the funnel because it had the highest prevalence of trismus in RCT vs. RT (OR = 9.82, 95% CI = 3.38-28.49). Egger’s test (*p* = 0.106) and Begg’s test (*p* = 0.211) did not show a significant risk of publication bias.

-Qualitative analysis of the outcomes related to the quality of life

Among the four articles that evaluated the quality of life and adverse effects of cancer treatment, none directly evaluated the impact of CRT vs. RT on quality of life. Associations were made based on the primary objectives of the studies and indirectly analyzed in this systematic review.

Cardoso *et al*., (2020) used MDASI-HN (MD Anderson Symptom Inventory- Head and Neck Module), EQ-5D (EuroQol- 5 Dimension) visual analogic, and MDADI (MD Anderson Dysphagia Inventory). These scales demonstrated that the greater the severity of trismus, the worse the quality of life.

Lee *et al*., (2014) assessing the HAD-depression scale (Hospital Anxiety and Depression Scale) and the EORTC QLQ-HN35 scale (European Organization for Research and Treatment of Cancer Quality of Life Questionnaire Head and Neck Cancer Module), noted that trismus patients showed worse scores of depression, social eating, social contact, sexuality, teeth, opening mouth, dry mouth, feeling ill, nutritional supplement and, weight loss.

Steiner *et al*., (2015) used a proprietary unvalidated visual analog scale measuring trismus-related discomfort from 0 to 10. He observed that QoL (quality of life) is inversely proportional to mouth opening.

**Figure 2:**
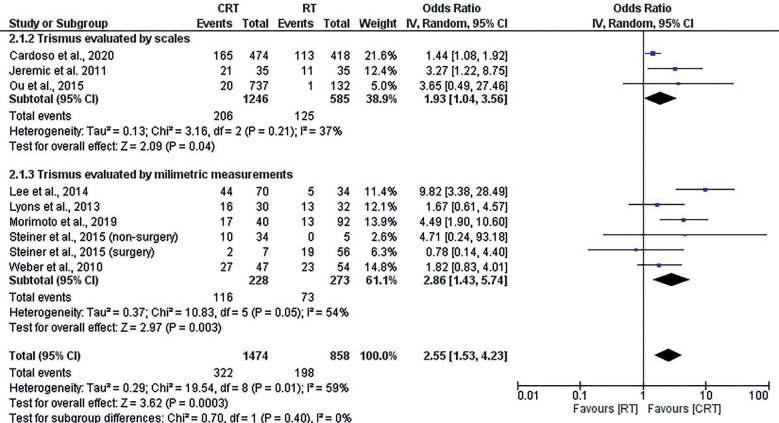
Forest plot from meta-analysis comparing incidence of trismus in RCT vs. RT head and neck patients.

**Figure 3:**
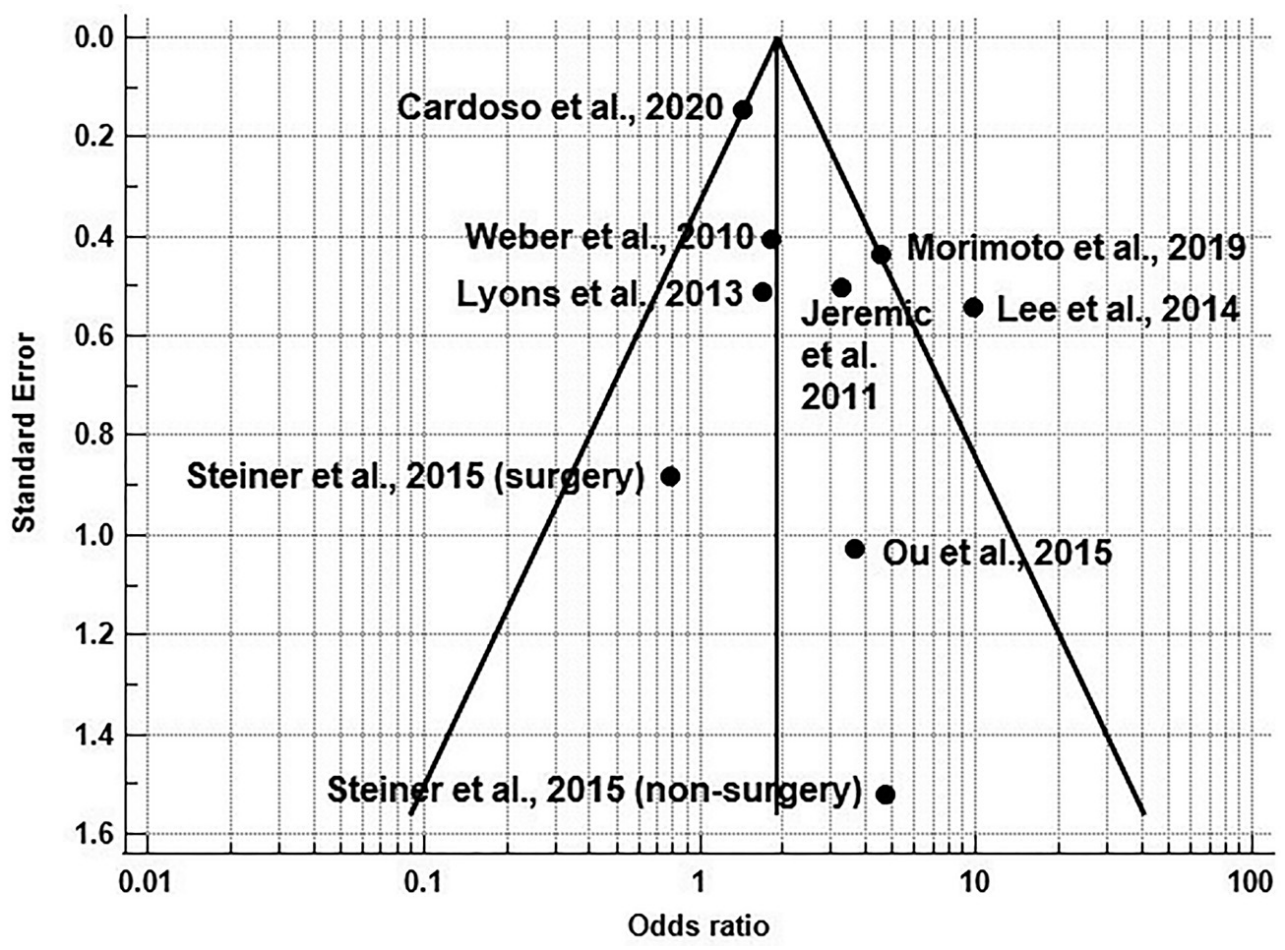
Funnel plot showing no significant risk of bias publication of meta-analysis comparing incidence of trismus in RCT vs. RT head and neck patients.

Weber *et al*., (2010) using the EORTC QOL (European Organization for Research and Treatment of Cancer Quality of Life Questionnaire Head and Neck Module (QLQ-H&N35), described that patients with tumors of the mouth and oropharynx have more adverse effects, more limitation of mouth opening, and poorer quality of life, (Figs. 2,3).

-Level of certainty of the evidence

GRADE analysis showed that the outcome of trismus incidence had a moderate level of certainty (Table 4).

**Table 4:**
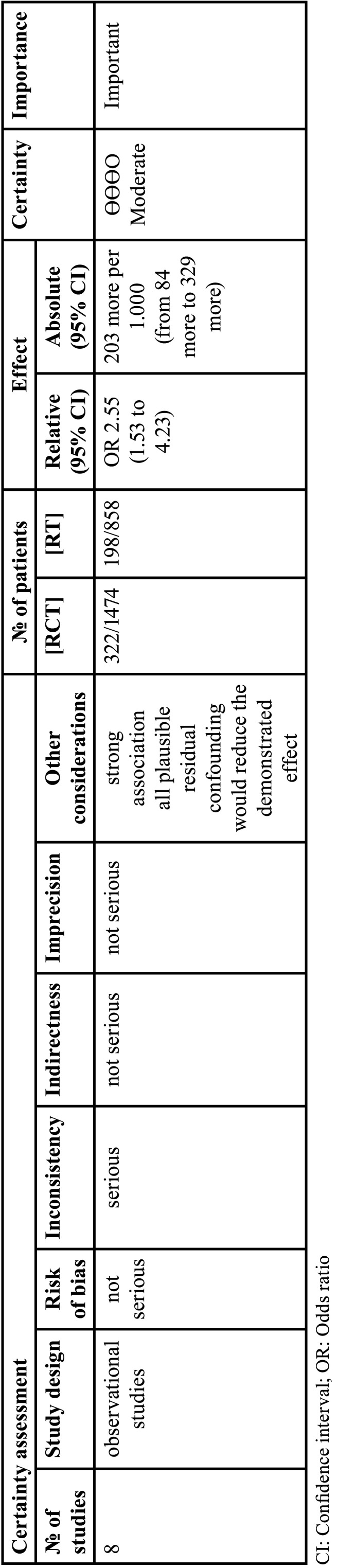
GRADE assessment of studies included in systematic review.

## Discussion

This systematic review demonstrated that CRT increases the prevalence of trismus in patients undergoing head and neck RT. Secondarily, it could also be qualitatively described that trismus is inversely related to worse quality of life. Trismus associated with RT and RCT is directly related to fibrosis and pain of the masticatory muscles, contracture in the masticatory structures (masseter, temporalis, and lateral and medial pterygoid muscles), direct damage to their innervation, and in some cases, degeneration of the temporomandibular joint (34). These changes can result in a considerable reduction in mouth opening, appearing soon after the onset of head and neck RT and last for months after treatment (35).

Dosimetry studies show a direct relationship between the radiation dose delivered on the masseter and medial pterygoid muscles (36). Apparently, average doses higher than 40 Gy are already able to generate significant damage and limitation of mandibular function (37). Although it is not possible to verify a direct relationship with the radiotherapy dose due to how the data are arranged, in this systematic review, the prevalence of trismus was significantly increased in patients treated with RCT, suggesting that chemotherapy may intensify this damage.

In a study by (38) that longitudinally evaluated the saliva of patients with head and neck cancer undergoing treatment with RT and CRT, was showed an increase in cytokines IL-1β, IL-6 and TNFα in the saliva of patients who underwent CRT compared to RT (38,39). In our study, we observed an increase in cases of trismus in patients undergoing CRT, we suggest that this increase in the incidence of trismus is due to the increase in the inflammatory profile that treatment with CRT induces, reducing the possible repair of these muscle cells, leading to progressive fibrosis.

Patients with head and neck tumors in more advanced stages commonly undergo RT treatment with CT concomitantly, and the risk of trismus may be linked to the advanced stages of the tumors. However, studies have shown that tumor staging is not independently related to the risk of developing trismus (31,32). Steiner (33), one of studies included in meta-analysis, addresses another confounding factor, showing that patients who have undergone previous surgery have a higher risk of developing trismus when compared to patients who only undergo RT. In addition, it does not show significant statistical difference related to mouth opening reduction between patients treated with RT + Surgery and Surgery, but we reveal a significant statistical difference of these groups when compared to patients who undergo CRT + Surgery, which is a profile of patients with the greatest risk of developing trismus.

In a systematic review, (40) described that the prevalence of trismus in irradiated patients is 44.1% at six months and decreased to 32.1% at 12 months and continued in average 32.6% at 3-10 years. This sequela, therefore, demonstrates a characteristic of starting acutely and continuing chronically over long periods. As RCT increases this prevalence, it is expected that the time course of trismus will also be extended.

RT-associated trismus is considered dose-dependent, and doses delivered to muscles such as Masseter and Pterygoid are important predictors for this condition (41). In a systematic review of 22 studies, (42) reported the prevalence of trismus after RT to be 25.4% for conventional RT, 5% for IMRT, and 30.7% for a combination of RT and chemotherapy. Although an accurate analysis on RT modalities and the impact of chemotherapy in each modality was not possible due to the absence of data specifying the subtypes of radiotherapy treatment, (27) also described that trismus is more incident in 3D-RTC than IMRT.

Platinum-based chemotherapies are the first lines of treatment for head and neck tumors, and cisplatin demonstrates significant clinical benefits compared to carboplatin. Cisplatin associated with RT demonstrates better prognosis and fewer adverse effects and is preferentially used (1). Part of the adverse effects of cisplatin is associated with its inflammatory potential. Cisplatin increases the systemic expression of several inflammatory cytokines overloading the liver and kidneys (22). As masticatory muscles are highly susceptible to the inflammatory process and trismus a strongly inflammatory consequence (43), systemic inflammation promoted by chemotherapy likely intensifies the damage to irradiated muscle tissue. Unfortunately, the description of protocols for chemotherapy is poorly described in the surveyed articles. Only two papers mention cisplatin (30,31) and one mentions cetuximab, making it difficult to speculate on clearer mechanisms involved in the increased prevalence of this adverse effect.

Being strongly related to difficulty chewing, swallowing, and speaking, trismus strongly impacts the quality of life. Four of the studies surveyed assessed quality of life and demonstrated an association between trismus and deficits in QoL (16,27,29,33). This relationship is associated with a reduction in the ability to work and attend leisure, social and family, and more problems according to physical function, pain, and loss of appetite (clinically significant), increasing the incidence of anxiety and depression post antineoplastic treatment (16,21) reported the negative impact on QoL reported by patients irradiated for oropharyngeal cancer treatment due to speech impairment, voice change, taste change, chewing problems, impaired swallowing, choking on food, and coughing when eating. The papers did not allow for a direct association between QoL in RCT vs. RT patients, but since the prevalence of trismus is higher in RCT patients, a worsening QoL is expected in these patients.

Trismus also makes eating difficult (29) demonstrated an inverse correlation between mouth opening and body mass index in head and neck irradiated patients (β = -0.33, *p* <0.005), with radiation doses being directly related to this process (r = 0.60, *p* <0.001). Due to the impact on masticatory function (44) and food intake (45), RT-associated trismus worsens overall survival in irradiated head and neck cancer patients, significantly reducing five-year overall survival in these patients (46).

An additional finding of this review revolves around the methodologies for assessing trismus. Methodologies using metric scales were superior to methodologies using subjective scales or rating systems. This finding suggests that further studies should adopt metric measures over semi-quantitative modalities.

Perhaps the most significant limitation of this review is that no study aimed to compare CRT and RT. This was not the primary endpoint of any of the included studies, which naturally made it difficult to create more subgroup analyses, made direct meta-analysis with the quality of life instruments impossible, and made it difficult to extract data for meta-analysis of the primary endpoint (trismus). However, the low risk of individual bias in most studies and moderate to low risk of collective bias, the low risk of publication bias, the moderate certainty of the evidence, and the low level of heterogeneity among studies demonstrate that additional studies will probably modify little the primary outcome found.

Hence, despite these limitations, this is the first paper that synthesizes this information concluding that the incidence of trismus increases when systemic chemotherapy is combined with head and neck radiotherapy. Since trismus impacts the overall survival of head and neck cancer patients (46), the cost of treating RT-induced trismus is extremely high (47). Furthermore, there are no effective therapeutic protocols to treat RT-related trismus (48). well-designed clinical trials should be suggested to outline methodologies to prevent this adverse effect.
